# Mass spectrometry-based proteomic analyses of contact lens deposition

**Published:** 2008-02-08

**Authors:** Kari B. Green-Church, Jason J. Nichols

**Affiliations:** 1 Campus Chemical Instrument Center Mass Spectrometry & Proteomics Facility; 2 College of Optometry, The Ohio State University, Columbus, OH

## Abstract

**Purpose:**

The purpose of this report is to describe the contact lens deposition proteome associated with two silicone hydrogel contact lenses and care solutions using a mass spectrometric-based approach.

**Methods:**

This was a randomized, controlled, examiner-masked crossover clinical trial that included 48 participants. Lenses and no-rub care solutions evaluated included galyfilcon A (Acuvue Advance, Vistakon Inc., Jacksonville, FL), lotrafilcon B (O2 Optix, CIBA Vision Inc., Duluth, GA), AQuify (CIBA Vision Inc.), and ReNu MoistureLoc (Bausch and Lomb Inc., Rochester, NY). After two weeks of daily wear in each lens-solution combination, the left lens was removed by the examiner (using gloves and forceps) and placed in a protein precipitation buffer (acetone). The precipitate was quantitated for total protein concentration (per lens), and proteins were then identified using liquid chromatography tandem mass spectrometry (nano-LC-MS/MS) and peptide sequencing.

**Results:**

Between 7.32 and 9.76 µg/lens of protein was observed on average from each lens-solution combination. There were 19 total unique proteins identified across the two lens materials, and six proteins were identified in all four lens-solution combinations including lipocalin, lysozyme, lacritin, lactoferrin, proline rich 4, and Ig Alpha. Lotrafilcon B was associated with 15 individual proteins (across both care solutions), and 53% of these proteins were observed in at least 50% of the analyses. Galyfilcon A was associated with 13 individual proteins, and 38.5% of these proteins were observed in at least 50% of the analyses. There were three unique proteins identified from galyfilcon A and four unique proteins identified from lotrafilcon B.

**Conclusions:**

The total amount of proteins identified from silicone hydrogel materials is much less than the amount from traditional soft lens materials. For the most part, the deposition proteome across these lenses is similar, although the different polymer characteristics might be associated with some variability in observance of the less frequently identified proteins.

## Introduction

Deposition of tear film components on contact lenses has been known for many years. Deposition is typically thought of as being different from a biofilm or pellicle. A bioflim or pellicle is a normal physiologic coating over the surface of a biomaterial whereas deposition should be considered a more significant and potentially pathological finding on a contact lens [1,2]. Short-term deposition-related complications may be relatively harmless with occasional symptoms of discomfort or visual disturbances [3,4]. Longer-term deposit-related complications could impact ocular health (for example, inflammatory responses such as contact lens papillary conjunctivitis) [5-8].

Deposition occurs across lens materials despite attempts to improve cleaning regimens or modify polymer materials and wear schedules. The quantity and composition of contact lens deposition is influenced by the material characteristics based on the Food and Drug Administration (FDA) categorization and species of interest (for example, lipid, protein, or inorganic). In terms of protein deposition, it is generally recognized that FDA group IV materials deposit the greatest amount of protein (up to approximately 1,000 μg/lens) while FDA Group I materials deposit the least protein (up to approximately 10–20 μg/lens) [9-17]. Ionic and higher water content materials tend to attract more protein than nonionic materials [10,14,15,17-23]. Recent work on silicone hydrogels has shown that these lenses deposit up to about 10 μg per lens, although this has not been extensively studied ex vivo [24-29].

In terms of the composition of protein deposition associated with contact lens materials, most of the work has centered around lysozyme using chromatographic or assay based methods [12,16,20,24,26,27,30-34]. Initial work via mass spectrometry was done by matrix-assisted laser desorption ionization (MALDI) mass spectrometry, although identifications of each species in the proteome were not conducted [20,35,36]. Today’s more sophisticated analytical techniques are likely to change our idea of the contact lens-related deposition proteome present. In this regard, contemporary analytical techniques could provide a more comprehensive understanding of the deposition proteome across polymer materials, care solutions, and wear schedules. The primary aim of this work was to use a mass spectrometry-based approach to evaluate the contact lens deposition proteome associated with two daily wear silicone hydrogel contact lenses when used with two multipurpose care solutions.

## Methods

### General study design and patient sample

This was a randomized, controlled, examiner-masked, crossover clinical trial. The protocol was approved by the University Institutional Review Board according to the tenets of the Declaration of Helsinki, and all subjects gave their informed and written consent. Following consent, study criteria were reviewed with each subject. Subjects were required to be 18 years of age or older, have 20/40 visual acuity with habitual contact lenses, have “healthy” eyes (taking no ocular medications), have a refractive error between –1.00DS and –6.00DS (and < 1.00 DC), and be a current silicone hydrogel lens wearer (daily wear only, seven days per week, 12 h per day for at least one month).

The two lenses used in this study were galyfilcon A (Acuvue Advance, Vistakon Inc., Jacksonville, FL) and lotrafilcon B (O_2_ Optix, CIBA Vision Inc., Duluth, GA), and the two care systems used in the study were AQuify (CIBA Vision Inc.) and ReNu with MoistureLoc (Bausch and Lomb Inc., Rochester, NY). Table 1 provides details of the composition of each material and lens care solution. All lens and care solution combinations (i.e., four treatment combinations given the number of lens-solution combinations) were randomly assigned to subjects who wore the lenses on a daily basis for 14–17 days and used the care solutions as indicated by their respective manufacturer (i.e., no rub, rinse only). Subjects returned after each combination for an outcome visit when contact lenses were removed as described below.

**Table 1:** Components of contact lens polymers and care solutions used in the study.

**Device or Care Solution**	**Composition**
AQuify Multi-Purpose Care Solution	Sorbitol
	Tromethamine
	Pluronic F127
	Sodoum phosphate dihydrogen
	Dexpanthnol
	Edetate disodium diohydrate
	Polyhexanide 0.0001
ReNU MoistureLoc Care Solution	Alexidine
	Borid acid
	Sodium chloride
	Sodium phosphate
	Hydranate
	Tetronic 1107
	Poloxamer 407
	Polyquarternium 10
Acuvue Advance (contact lens)	Monofunctional polydimethylsiloxane (MPDS)
	“N,N-dimethylacrylamide (DMA)”
	Ethyleneglycol dimethacrylate (EGDMA)
	Poly-2-hydroxyethyl methacrylate (pHEMA)
	Polyvinyl pyrrolidone (PVP)
	Siloxane macromer
O2 Optix (contact lens)	“N,N-dimethylacrylamide (DMA)”
	Tris-(trimethylsiloxysilyl) propylvinyl carbamate
	Siloxane macromer

### Lens collection and sample pooling

The masked examiner wore latex-free gloves and used ophthalmic tweezers to remove the lenses from the eyes of subjects. Lenses from the left eyes of subjects were then placed directly in a protein precipitation buffer for subsequent proteomic analysis (described below). Lenses were not rinsed before storage. Six individual subject samples were pooled in composites within each treatment combination (lens-solution combination). Thus, for each lens-solution treatment combination (for which all 48 subjects experienced), a set of eight composites was obtained for analyses.

### Protein precipitation from contact lenses and quantitation

Each sample was mixed with acetone at –20 °C at a ratio of 1:10 sample/acetone and precipitated overnight at –20 °C. This was then centrifuged at room temperature for 10 min, the supernatant was removed, and it was repeated a second time. The samples were allowed to air dry for approximately 15 min before re-suspending them in water.

The proteins were quantitated using the Bradford Assay (reported in µg/lens for each lens-solution combination). A stock solution of BSA (250 µg/ml or 500 µg/ml) using Pierce BSA stock solution was prepared in water, and 30 µl of the BSA solution was diluted into 270 µl of Bradford dye reagent and mixed. The mixture (25 ng/µl BSA) was then allowed to equilibrate for 10 min and was used to create a standard curve. A blank reading using a spectrophotometer was obtained using 90 µl of Bradford reagent at an absorbance of 595 nm. Next, 10 µl of the BSA/Bradford dye solution was added to the Bradford reagent and thoroughly mixed, and the absorbance was read. This was repeated at least 10 more times to generate a set of concentration standards and a calibration curve.

Sample (1 μl) and 9 µl of water were added to 90 µl of Bradford reagent and allowed to equilibrate for 10 min. The sample absorbance was read to the second decimal place using the spectrophotometer, and the process was repeated two more times. The calibration curve data was used to calculate the sample concentration, and the protein concentration was calculated by multiplying the dilution factor used for the assay. This method is generally linear up to 15 µg/ml, and the minimum accurate concentration reading is approximately 1 µg/ml. The final protein concentration is reported in µg/lens for each lens-solution combination.

### Trypsin digestion and protein identification

Protein (5 µg) from each pooled composite was digested using trypsin [37]. Nano-liquid chromatography tandem mass spectrometry (nano-LC-MS/MS) was performed on a Thermo Finnigan LTQ mass spectrometer. The LC system was an UltiMate™ Plus system from LC-Packings A Dionex Co. (Sunnyvale, CA). The scan sequence of the mass spectrometer was based on the TopTen™ method using dynamic exclusion. Sequence information from the MS/MS data was processed by converting the raw data (.dta) files into a merged file (.mgf) using MGF creator (merge.pl, a Perl script). The resulting mgf files were searched using Mascot Daemon by Matrix Science (Boston, MA). Protein identification was confirmed on a minimum of two sequenced tryptic peptides with a minimum string of five amino acids [38,39]. Assigned peaks had a minimum of 10 counts (signal:noise of three). The mass accuracy of the precursor ions was set to 1.8 Da and the fragment mass accuracy was set to 0.5 Da. Considered modifications (variable) included methionine oxidation and carbamidomethyl cysteine.

### Statistical analyses

Protein quantitation results were compared across treatment combinations using nonparametric ANOVA (Friedman’s test) with Dunn’s multiple comparison post-hoc test as appropriate. Protein identification results were tabulated, whereby the frequency of each individual protein was determined across the eight composites within each combination. An arbitrary a priori decision to include frequently observed proteins was based on the number of composites (out of eight) in which the unique protein was identified. In this regard, a protein was considered to be frequently observed if it was observed in at least 50% of the composites.

## Results

### Protein quantitation

Due to a technical difficulty, the first composite of eight lenses was not able to be quantitated due to an inefficient extraction complication (although information about protein identification was obtained from this composite). There were different protein concentrations across lens-care solution combinations (Friedman’s statistic=9.00, p=0.02). Galyfilcon A was generally associated with slightly less total protein than lotrafilcon B regardless of care solution (1.71 µg/lens less on average than lotrafilcon B). Galyfilcon A lenses showed an average protein quantitation of 8.78 **±** 1.49 µg/lens (median=8.70 µg/lens) with AQuify and 7.32 **±** 0.90 µg/lens (median=7.45 µg/lens) with ReNu MoistureLoc. Lotrafilcon B lenses showed an average protein quantitation of 9.75 **±** 1.43 µg/lens (median=10.00 µg/lens) with AQuify and 9.76 **±** 0.96 µg/lens (median=10.01 µg/lens) with ReNu MoistureLoc. Post-hoc testing revealed significantly less total protein content in galyfilcon A when compared to either lotrafilcon B with AQuify or lotrafilcon B with Renu (both p<0.05); no other post-hoc comparisons differed significantly.

### Protein identifications (nano-LC-MS/MS)

Table 2 lists the proteins identified from the eight composites within each lens-solution combination (again, each was a pooled sample of six lenses). Overall, 13 proteins were identified from galyfilcon B while 15 proteins were observed from lotrafilcon B. The following six proteins were identified in all four lens-solution combinations of the trial (reported by relative frequency of observance in terms of the number of composites in which the protein was observed): lipocalin (the most frequently observed), lysozyme, lacritin, lactoferrin, proline rich 4, and Ig Alpha. The following three proteins were identified in three of the four lens-care solution combinations of the trial (reported by relative frequency of observance in terms of the number of composites in which it was observed): Ig Kappa, secretoglobin 2A1, and prolactin induced protein.

**Table 2:** Protein identifications and the frequency of observance of the protein relative to the eight composites analyzed per combination.

Frequency (out of 8 composites)	Acuvue Advance & AQuify	Acuvue Advance & ReNu	O2 Optix & Aquify	O2 Optix & ReNu
8		Lipocalin (LCN1)		Lipocalin (LCN1)
7			Lipocalin (LCN1)	Lacritin (LACRT)
6			Lysozyme (LYZ)	Lysozyme (LYZ)
5	Lipocalin (LCN1)	Lysozyme (LYZ)	Lactoferrin (LTF)	Proline Rich 4 (PRR4)
	Lysozyme (LYZ)		Proline Rich 4 (PRR4)	
4		Lactoferrin (LTF)	Ig Alpha (CD79A)	Ig Kappa (IGKC)
		Lacritin (LACRT)	Secretoglobin 2A1 (SCGB2A1)	Lactoferrin (LTF)
		Ig Alpha (CD79A)	Lacritin (LACRT)	
3	Lactoferrin (LTF)		Cystatin SN (CST1)	Prolactin Induced Protein (PIP)
	Lacritin (LACRT)			Secretoglobin 2A1 (SCGB2A1)
	Proline Rich 4 (PRR4)			
2	Ig Alpha (CD79A)	Secretory Component (ECM1)	Prolactin Induced Protein (PIP)	Ig Alpha (CD79A)
		Proline Rich 4 (PRR4) Secretoglobin 2A1 (SCGB2A1)		
		Prolactin Induced Protein (PIP)		
1	Ig Kappa (IGKC)	Carbonly Reductase (CBR1)	Secretoglobin 2A2 (SCGB2A2)	Secretoglobin 2A2 (SCGB2A2)
	Secretoglobin 2A1		Secretoglobin 1D1 (SCGB1D1)	Secretoglobin 1D1 (SCGB1D1)
	(SCGB2A1)		Ig Kappa (IGKC)	Ig Lambda (IGLC2)
	Secretory Component (ECM1)		Zn alpha 2 glycoprotein (AZGP1)	Carbonyl Reductase (CBR1)
	Basic Proline Rich (PROL1)			
	Heat shock Protein 27 (HSPBAP1)			
Total proteins per arm	11	11	13	13
Total unique proteins per material	13		15	

Note that the gene symbol for each protein is provided in parentheses. Secretoglobin 2A1 is also known as mammaglobin B. Secretoglobin 2A2 is also known as mammaglobin A. Secretoglobin 1D1 is also known as lipophilin A.

When evaluating proteins extracted from the ReNu combinations of the trial, lipocalin was observed in all eight composites for both galyfilcon A and lotrafilcon B while none of the proteins were observed in all eight composites when evaluating those associated with AQuify. When AQuify was used with galyfilcon A, lipocalin was identified in five of the eight composites, and when AQuify was used with lotrafilcon B, lipocalin was observed in seven of the eight composites. Lotrafilcon B lenses with either care solution (AQuify or ReNu) were associated with a total of 15 individual proteins observed over all composites, and eight (53.3%) of these proteins were observed in at least four (50%) of the composites. Galyfilcon A lenses were associated with a total of 13 proteins (again, regardless of the care solution), and five (38.5%) of these proteins were observed in 50% of the composites. There were two unique proteins identified from galyfilcon A only (basic proline rich protein and heat shock protein), and four unique proteins were identified from lotrafilcon B only (secretoglobin family 2A member 2, Ig Lambda, Zn alpha 2 glycoprotein, and secretoglobin 1D1).

## Discussion

To our knowledge, this work represents one of the first reports of the use of mass spectrometry (specifically nano-LC-MS/MS and bioinformatics) to explore the contact lens-related proteome. Gel chromatography or assay based methods have traditionally been used when evaluating proteins associated with contact lenses, and much work has centered primarily on lysozyme [17,19,20,24,26,27,30-33,36,40-46]. However, recent reports using mass-spectrometry based methods have shown the number of proteins associated with the tear film range into the hundreds [47-49]. Certainly, lysozyme and other traditionally understood proteins such as lactoferrin, lipocalin, and albumin continue to be identified in the tear film (as most were here), but the extent of our knowledge of the tear film proteome has lead to new insights into fundamental processes and proteins present both in health- and disease-related states. It is important that we also extend our knowledge and fundamental understanding of the proteome typically associated with hydrogel lens wear to further identify the role these proteins may have in the continued safe, comfortable wear of contact lenses.

These results suggest that there were six proteins observed across all material/care solution treatment combinations in this trial (lipocalin, lysozyme, lacritin, lactoferrin, proline rich 4, and Ig Alpha), and there were another three that were observed in three of the four lens-care solution combinations (Ig Kappa, secretoglobin 2A1, and prolactin induced protein). Thus, these nine proteins appear to make up the bulk of the contact lens-related proteome associated with these two silicone hydrogel materials. It should be noted that these results are relevant to this particular precipitation buffer (acetone); in this regard, the use of any buffer may differ for different lens materials. This is likely not the case though for the small differences in protein quantitation found here but may be associated with differences in the infrequently observed unique proteins.

It was also observed that the more frequently observed (possibly higher abundant) proteins from both lens material types seem to be fairly consistent as the same proteins were generally observed regardless of the different care solutions for each lens material. This is true even though the care solutions differ in terms of their composition, although differences in their composition may be related to the identification of some of the more infrequently observed proteins. The lotrafilcon B material with either care solution was associated with a total of 15 unique proteins observed over all analyses with eight (53.3%) of these proteins being observed in at least one half of the analyses. Similarly, galyfilcon A lenses were associated with a total of 13 unique proteins (again, regardless of the care solution), and five (38.5%) of these proteins were observed in one half of the analyses. The five commonly observed proteins (i.e., observed at least 50% of the time for both material types) were lipocalin, lysozyme, lactoferrin, lacritin, and Ig Alpha. The proteome observed from the lotrafilcon B lenses showed slightly more diversity in the frequency of the observed proteins (i.e., observed more than 50% of the time but only from the lotrafilcon material) and also included proline rich 4, secretoglobin 2A1, and Ig Kappa. As these proteins are commonly observed in the tear film, it could be that these proteins are more strongly bound to the galyfilcon A material thus more difficult to extract from the lens; alternatively, they may not have an affinity for galyfilcon A itself, although this seems unlikely. Although lotrafilcon B was associated with slightly higher total quantities of proteins than the galyfilcon A lenses (~1.7 µg/lens), it is highly unlikely that this difference has any clinical relevance. It could be that lotrafilcon B inherently binds more proteins than galyfilcon A or that both materials have a similar affinity for tear film proteins, but galyfilcon A binds the proteins more strongly, making their removal more difficult with a no-rub multipurpose care solution. It is not likely that there are differences in the efficacy of the two care solutions as very similar levels of total proteins were extracted within each material. However, it is important to emphasize that the total protein quantities from each lens materials, nor the differences between the lens materials, are not likely associated with any clinical impact on lens performance (i.e., fitting characteristics, visual performance, or subjective outcomes). This is particularly true for FDA Group IV lenses as they are known to deposit massive amounts of total protein (up to 1,000 µg/lens), yet these lenses have been clinically used quite successfully for years. Rather, the identification of individual proteins associated with these contact lenses may lend itself to a broader fundamental understanding of the impact of contact lenses on ocular surface/tear film physiology itself. While clinical outcomes could be related to the total protein quantity, another consideration is the conformational state of the proteins (such as tertiary versus denatured), which was not studied in the present work [50].

There were also some unique proteins identified from both galyfilcon A and lotrafilcon B (although infrequently observed). For instance, heat shock protein 27 (associated with thermal upregulation associated with stress response [51]), basic proline rich protein (with anti-viral, among other properties [52,53]), and the secretory component were associated with galyfilcon A. Interestingly, the secretory component was associated with galyfilcon A with both care solutions, although this was not associated with lotrafilcon B. Likewise, cystatin S1 (cysteine protease inhibitor [54]), Ig lambda, and secretoglobin 1D1 were associated with lotrafilcon B but not galyfilcon A. It is important to note that lenses were not rinsed following removal from the eye, which might remove the more extraneous proteins that are not necessarily bound to the material itself. This certainly could impact the proteins that are observed, in particular for the aforementioned less commonly observed proteins. In this regard, some proteins that are associated with the lens but not necessarily bound to it may be observed due to things like the lens removal process itself, surface area differences, surface tension differences, or other extraneous factors; however, if this were the case, it would seem that we would identify numerous proteins given the vast quantity of proteins identified in the tear film itself. There was no adaptation period for subjects entering the study, and each was required to have at least one month of silicone hydrogel lens wear. Thus, it could be possible that experience with new lens materials lead to differences in proteins that were expressed (or not expressed). However, the approach used probably allows the most global assessment of the entire lens-related proteome as not all patients rinse their lenses before storage. Thus, the approach presented here might be reflective of the proteome associated with this clinical situation.

In summary, the quantities of proteins associated with silicone hydrogels are fairly minimal compared to traditional soft lens materials. While there are small differences, it is unlikely that these differences impact clinical performance. The contact lens deposition proteome appears to be fairly consistent between these two silicone hydrogels which consist mainly of six proteins including lipocalin, lysozyme, lacritin, lactoferrin, proline rich 4, and Ig Alpha. However, unique proteins were observed from each polymer type, and while it is not expected that these differences would have an impact on the clinical level per se, they do yield insight into the tear film proteome itself as well as the way in which these polymers interact with the tear film proteome.
